# Integrating HIV Into Sexual and Reproductive Health Services for Female Sex Workers in Buenos Aires, Argentina: A Comprehensive Model Designed Through Intersectional Collaboration

**DOI:** 10.1002/jia2.70167

**Published:** 2026-07-25

**Authors:** María Macarena Sandoval, Inés Aristegui, Virginia Zalazar, Silvina Vulcano, María Victoria Iannantuono, Agustina Argüello, Emilia Frontini, Ana C. Zeltman, Agustín Nava, Nadir F. Cardozo, Marcela Romero, Georgina Orellano, Carolina F. Pérez, María Inés Figueroa, Adriana Durán, Sharon Walmsley, Valeria Fink, the “MAS por Nosotras” study team

**Affiliations:** ^1^ Research Department Fundación Huésped Buenos Aires Argentina; ^2^ Coordination of Sexual Health, HIV, Sexually Transmitted Infections and Tuberculosis of the Ministry of Health Government of the City of Buenos Aires Buenos Aires Argentina; ^3^ Asociación De Travestis Transexuales y Transgénero de Argentina Buenos Aires Argentina; ^4^ Sindicato De Trabajadorxs Sexuales De Argentina Buenos Aires Argentina; ^5^ University Health Network, University of Toronto Toronto Ontario Canada

**Keywords:** HIV, Latin America, primary prevention, reproductive health, sex workers, women

## Abstract

**Introduction:**

Female sex workers (FSWs) are at higher risk of sexually transmitted infections (STIs), including HIV, due to occupation‐related factors further exacerbated by gender‐specific vulnerability and marginalization. Stigma and fear of disclosing their occupation pose multiple barriers that limit access to sexual and reproductive health (SRH) services, as well as HIV testing, prevention and treatment. We describe the implementation of a tailored comprehensive SRH‐HIV prevention and care model for FSWs, and report HIV prevalence, pre‐exposure prophylaxis (PrEP) uptake and HIV treatment outcomes in our cohort.

**Methods:**

“MAS por Nosotras” adopted a co‐creation and co‐production approach including a formative phase (focus groups with FSWs and semistructured interviews with stakeholders), and a prospective cohort of FSWs at a non‐governmental organization (NGO) in Argentina. Our conceived model focused on HIV treatment and combination prevention, including STI testing, cancer screening, contraception counselling and vaccination. Demographic, psychosocial and clinical information were collected.

**Results:**

Between June 2023 and March 2024, 200 cisgender and transgender FSWs were enrolled in the prospective cohort, with a 66.5% retention rate at 6 months. HIV prevalence was 18.5% (34.3% in transgender women [TGW] and 3% in cisgender women [CGW]), with only one incident case during follow‐up. Among those with known HIV, 61% had a viral load <50 copies/mL. PrEP acceptance was higher in TGW (55.6%) versus 30.5% in CGW, but PrEP persistence was low (69% discontinued), especially in CGW. Nearly 78.5% of participants indicated they would be very likely to recommend the care package to their peers.

**Conclusions:**

“MAS por Nosotras” implemented a community‐linked, gender‐informed integrated SRH‐HIV model of care, articulated with community‐based organizations, public health and an NGO research centre that facilitated access to HIV testing, prevention and treatment initiation or re‐engagement. We found a high prevalence of HIV, particularly among TGW, and low PrEP knowledge and use, especially among CGW. Our data reflect the need of expanding and tailoring HIV prevention strategies with a community‐focused approach. This proposed integrated SRH‐HIV model addressed these needs and generated preliminary evidence regarding operational feasibility and acceptability that may inform models to help close prevention and treatment gaps for FSWs in Latin America.

## 1 Introduction

In Latin America, women are disproportionately affected by the intersecting inequalities related to age, gender, ethnicity, migration, sexual orientation, socio‐economic level, education and occupation. Female sex workers (FSWs) face some of the greatest barriers to realizing their basic health rights [1]. Although sex work is not illegal in Argentina, it is criminalized in practice [2, 3, 4], exposing FSWs to police and gender‐based harassment, such as sexual and physical violence [2, 3, 4, 5], precarious working conditions [6, 7], and economic and psychosocial vulnerability. These conditions limit access to sexual and reproductive health (SRH) services, and contribute to a high burden of physical and mental health problems [7, 8].

FSWs are vulnerable to sexually transmitted infections (STIs), including HIV, due to work‐related factors such as early sexual debut, multiple sexual partners and pressure to engage in condomless intercourse. High STIs and HIV prevalence among FSWs, exceeding general population rates, has been widely reported [9, 10, 11, 12, 13, 14], especially in transgender women (TGW) [15, 16, 17]. Globally, the relative risk of acquiring HIV for sex workers is nine times higher than for the general population [18], and the global median HIV prevalence among sex workers is 3% (range: 0%−62% [19]).

Argentina offers universal access to healthcare, including combined HIV prevention with HIV/STI testing and treatment provided free of charge. However, the HIV epidemic remains concentrated among key populations: TGW (34%), men who have sex with men (12%–15%) and FSWs (2%–5%) have the highest prevalence rates [10, 15, 20]. Service provision varies widely, and existing strategies often fail to meet the SRH needs of FSWs. Integrated care approaches are uncommon, requiring individuals to attend multiple consultations across different facilities to obtain contraception and family planning counselling, cervical and breast cancer screening, and other SRH services. To our knowledge, no health services dedicated to FSWs currently exist in the country.

Although Buenos Aires has longstanding public HIV testing and treatment programmes, the oral pre‐exposure prophylaxis (PrEP) programme did not begin until late 2021, with scarce information about PrEP among FSWs.

“MAS por Nosotras” emerged as a multicomponent project to address the SRH of FSWs in the metropolitan area of Buenos Aires. Building on lessons learned during the COVID‐19 pandemic—which deepened pre‐existing barriers to care in this population—the project sought to establish a community‐linked, gender‐focused care model able to remain functional and adaptable during health emergencies. By integrating HIV and SRH services, the initiative aimed to ensure more sustainable support for marginalized populations.

The objective of this study is to describe the development and implementation of an integrated SRH‐HIV prevention and care model tailored for FSWs, and to report HIV prevalence, oral PrEP uptake and HIV treatment outcomes in our cohort.

## 2 Methods

### 2.1 The Project: “MAS por Nosotras”

“MAS por Nosotras” (“More for us,” aiming to empower the community to care for themselves), an initiative whose name derives from Modelo de Atención en Salud (Healthcare Delivery Model), was conducted at Fundación Huésped (FH), a non‐governmental (NGO) research organization in Buenos Aires, Argentina. The project was developed through a collaboration between FH, the local Ministry of Health (MoH), two community‐based organizations—*Sindicato de Trabajadorxs Sexuales* (AMMAR) and *Asociación de Travestis, Transexuales y Transgénero de Argentina* (ATTTA)—, and a Canadian research team (University Health Network, University of Toronto). This multi‐stakeholder partnership adopted a co‐creation and co‐production approach, including active involvement of peer research associates, peer navigators and community organizations, ensuring that the design, implementation and refinement of the model were grounded in the lived experiences and priorities of FSWs communities, thereby guaranteeing cultural relevance and acceptability.

**FIGURE 1:**
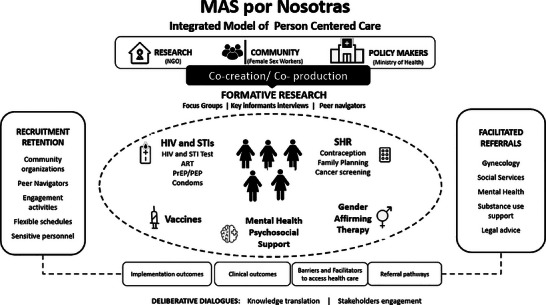
Integrated model of person‐centred care. Abbreviations: ART, antiretroviral therapy; HIV, human immunodeficiency virus; NGO, non‐governmental organization; PEP, post‐exposure prophylaxis; PrEP, pre‐exposure prophylaxis; SHR, sexual and reproductive health; STIs, sexually transmitted infections.

The project comprised formative research, a prospective cohort study and deliberative dialogues focused on stakeholder engagement and knowledge translation activities (Figure 1). The study protocol was approved by the Institutional Review Board (IORG0001557). Participants provided written informed consent and were economically compensated for their time and effort for research visits, covering transportation costs and lost income associated with attending the visit.

### 2.2 Formative Research

This qualitative component aimed to understand the needs of FSWs, access to SRH during the COVID‐19 pandemic, and to inform the establishment of the prospective cohort through community and stakeholder input. In December 2022, two focus groups, co‐facilitated by social researchers and peer research associates: one with transgender FSWs at ATTTA and one with cisgender FSWs at AMMAR, involved a convenience sample of 28 participants (15 transgender and 13 cisgender FSWs), recruited through the peer navigators and community‐based organizations. Focus groups explored barriers and facilitators to SRH‐HIV access, stigma and discrimination in healthcare settings, and health needs. Additionally, 33 key informant interviews were conducted with healthcare workers, government representatives and other stakeholders by trained social researchers between January 2023 and April 2024. Qualitative data were analysed using the DEPICT model [21]. Findings informed the design and procedures of the cohort study, and recommended package of services. As this formative phase was intended to guide model development and operational planning, rather than generate exhaustive qualitative findings, formal saturation was not prospectively assessed. Findings from focus groups, key informant interviews and ongoing stakeholder engagement were used iteratively to inform operational adaptations, including referral pathways and care navigation strategies.

### 2.3 Prospective Cohort

A prospective cohort of cisgender and transgender FSWs was created to pilot a gender‐focused integrated SRH‐HIV prevention and care model. This model included a package of services, consisting of HIV/STI testing, cancer screening, contraception, vaccination and medical evaluations. The cohort served as the platform to explore the operational feasibility and components of the integrated care model. While there are no official estimates of the number of sex workers in Argentina, a non‐probabilistic convenience sample of 200 participants, 100 cisgender women (CGW) and 100 TGW, was defined for this exploratory project, expecting to complete accrual in 6 months.

Participants were enrolled from June 2023 to March 2024. Eligibility criteria included being 18 years old or older; self‐identifying as CGW or TGW; identifying as a sex worker or a woman who had received money or goods in exchange for sexual services; and living in the metropolitan area of Buenos Aires with no plans to relocate during the study period. A unique identification number was assigned to each participant.

Each of two study visits, conducted 6 months apart, consisted of a comprehensive medical evaluation, combined HIV prevention, gynaecological and proctological assessments, contraception counselling, laboratory testing and STIs screening, and a structured psychosocial interview (Table 1), served the dual purpose of gathering epidemiological data and piloting the operational delivery of the model. Gender‐affirming hormone therapy was offered to transgender FSWs. If required, participants had additional medical consultations, vaccination or treatment delivery appointments. A complete description of the procedures performed during the visits is provided in Table S1. Data were captured using both paper and electronic case report forms (REDCap).

**TABLE 1:** Procedures performed at each visit.

Procedure	Baseline visit	6‐month visit	Additional visits (performed only when needed)
Informed consent	x		
Clinical evaluation	x	x	
HIV rapid test	x	x	x
Counselling and offer of PEP and PrEP	x	x	x
STI screening	x	x	x
STI counselling and offer of condoms	x	x	x
Laboratory tests	x	x	x
Vaccines	x	x	x
Gynaecological assessment	x	x	x
Proctological assessment	x	x	x
Hormone gender‐affirming therapy	x	x	x
Psychosocial interview	x	x	
Satisfaction and structural priorities assessment		x	
Psychological evaluation			x

*Note*: The “x” means that the procedure was performed in this visit.

Abbreviations: HIV, human immunodeficiency virus; PEP, post‐exposure prophylaxis; PrEP, pre‐exposure prophylaxis; STI, sexually transmitted infection.

### 2.4 Our Model of Care

The model was conceived as an integrated SRH‐HIV service delivery approach aligned with regional priorities for person‐centred care, using a “one‐stop, one‐visit” approach, to reduce structural barriers and offer key services. The formative research findings identified fragmented care and difficulties navigating multiple services as major barriers to SRH access.

As an exploratory pilot project, operational feasibility and acceptability were explored through predefined indicators including recruitment capacity, retention at follow‐up visits, participant satisfaction and willingness to recommend the service to peers.

### 2.5 Setting

“MAS por Nosotras” was implemented at FH, a facility with long‐standing experience in providing combined HIV prevention services in coordination with the MoH. Within the integrated SRH‐HIV model, FH provided HIV testing, counselling and linkage to antiretroviral treatment (ART), and offered post‐exposure prophylaxis (PEP) and PrEP through the programme already active at the site. The existing infrastructure, multidisciplinary team and community‐linked approach made FH a suitable platform for piloting the integrated model that included comprehensive SRH services for CGW. FH functioned as a primary SRH entry point, a role strengthened through SRH‐specific training provided by the MoH. All staff involved in services (administrative, medical and psychosocial personnel) received specific training in gender diversity, stigma reduction and trauma‐informed care as part of the co‐creation process with community partners.

To ensure continuity of care beyond the “one‐stop” visit, four referral pathways for secondary and tertiary public health services were coordinated with the MoH. These referral pathways, particularly for advanced gynaecological or pregnancy‐related care, were developed for the project by coordinating with existing public services and defining expedited routes for FSWs.

FH also offered existing on‐site psychosocial support when needed, including psychological counselling, legal orientation and case management. When participants required specialized mental health services or social programmes, they were formally referred to existing networks.

### 2.6 Recruitment and Engagement

Recruitment was conducted by three transgender peer navigators from the research team, alongside four cisgender community peer leaders from AMMAR and ATTTA. Communication materials and appointment reminders were developed to support recruitment and retention. Peer navigators played a central role in linkage to care, appointment coordination, accompaniment when needed and participant retention.

Community engagement was promoted throughout the project, with talks and workshops at the community‐based organizations. These activities foster interactions among members of FH, MoH, AMMAR and ATTTA, strengthening trust and collaboration.

As enrolment commenced and the aforementioned activities were conducted, the accrual accelerated, reflecting increasing confidence in the project and a possible snowball effect.

### 2.7 Statistical Analysis

All statistical analyses were performed using R (R Core Team, 2025) [22]. Descriptive statistics were used to summarize baseline characteristics, clinical outcomes, implementation indicators and behavioural variables. Continuous variables were reported as medians with interquartile ranges (IQRs), and categorical variables as absolute frequencies and percentages. There were no missing data for the variables included in the analyses.

Exploratory bivariate comparisons between TGW and CGW were performed to identify potential differences in health needs, services and outcomes that could inform the development of gender‐informed SRH‐HIV strategies, using the Wilcoxon rank‐sum test for continuous variables, and Pearson's chi‐squared test or Fisher's exact test for categorical variables. All statistical tests were two‐sided with a significance level set at *p* < 0.05.

## 3 Results

### 3.1 Summary Findings of Formative Research

The formative research highlighted three major domains impacting SRH‐HIV access among FSWs: anticipated stigma, competing structural priorities and reliance on community resources. Both CGW and TGW reported anticipating stigma within healthcare facilities. Although transgender‐specific healthcare services have improved over the past decade, persistent discrimination towards TGW continues to undermine their access to care. In addition, CGW feared facing judgement if they disclosed their occupation and frequently described competing priorities—such as childcare and daily income generation—that limited their ability to attend multiple daytime appointments. Participants highlighted the pivotal role of community resources, such as grassroots organizations, in mobilizing peer networks and disseminating information and support. These insights underscored the need for a low‐barrier, community‐linked model capable of consolidating services into a single visit, supported by sensitized healthcare workers to adequately attend to both cisgender and TGW.

### 3.2 Cohort Characteristics

**FIGURE 2:**
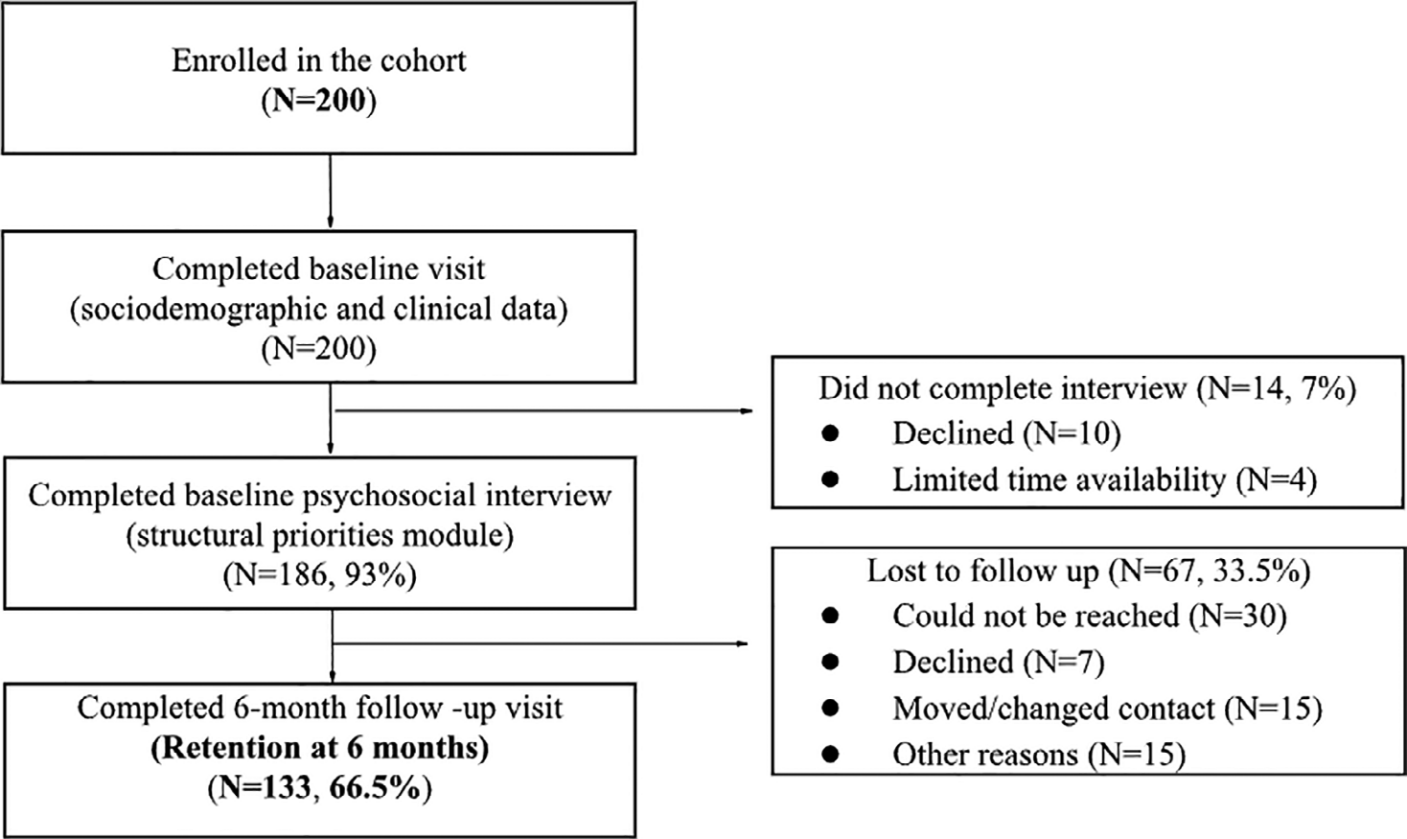
Flow diagram of the “MAS por Nosotras” cohort.

A total of 200 FSWs were enrolled (101 CGW and 99 TGW). All completed baseline medical evaluations, and 186 completed psychosocial interviews. The 6‐month follow‐up was completed by 133 participants (66.5% retention rate) (Figure 2).

Baseline characteristics are described in Table 2. Compared to CGW, TGW were younger, more likely to be internal migrants, had initiated sex work at an earlier age, reported a higher number of sexual partners and primarily worked on the street. Substance use and alcohol consumption were high across both groups.

**TABLE 2:** Baseline characteristics of the participants.

	TGW (*N* = 99)	CGW (*N* = 101)	*p*
Age: years (median [IQR])	29 (24−39)	36 (30−47)	*<0.001*
External migrant: *n* (%)	32 (32%)	28 (28%)	*0.5*
Internal migrant: *n* (%)	54 (81%)	15 (21%)	*<0.001*
Complete secondary school or higher education: *n* (%)	45 (48%)	48 (51%)	*0.7*
Lifetime tobacco use: *n* (%)	52 (52%)	71 (70%)	*0.035*
Lifetime substance use:			
Marijuana: *n* (%)	56 (63%)	57 (89%)	*<0.001*
Cocaine: *n* (%)	55 (62%)	37 (58%)	*0.6*
Lifetime alcohol consumption: *n* (%)	90 (90%)	88 (88%)	*0.3*
Age at sex work initiation: years (median [IQR])	18 (15−20)	23 (20−30)	*<0.001*
Years on sex work: years (median [IQR])	12 (6.5−19)	10 (5−19)	*0.5*
>20 sexual partners in the last month: *n* (%)	43 (44.8%)	21 (22.8%)	*<0.001*
Condomless anal or vaginal intercourse in the prior month: *n* (%)	57 (57.6%)	53 (52.5%)	*0.5*
Street as work venue: *n* (%)	74 (79%)	31 (31%)	*<0.001*
Sex work as main source of income: *n* (%)	87 (95%)	82 (87%)	*0.083*

Abbreviations: CGW, cisgender woman; IQR, interquartile range; TGW, transgender woman.

### 3.3 Structural Priorities at Follow‐Up

To better understand how gender‐based structural inequalities shape FSWs’ ability to access healthcare, participants were asked at the 6‐month follow‐up to select their top five priorities from a predefined list. This item was added following initial recruitment and retention challenges based on feedback from community leaders who suggested that difficulties in engaging in SRH care may reflect competing structural demands rather than a lack of interest.

Safe housing was identified as the most urgent need (rank first for 35.2% of participants and second for 13.9%). Access to dignified work—including sex work and other employment options—consistently appeared in third (19.8%), fourth (14.2%) and fifth (18.5%) positions, alongside economic stability, caregiving responsibilities and basic needs like food security. Although 15.6% of participants selected SRH as their highest priority, it was rarely mentioned in subsequent rankings.

### 3.4 HIV Prevalence and Incidence

Overall, HIV prevalence was 18.5% (*n* = 37), with 34.3% (*n* = 34) in TGW and 3% (*n* = 3) in CGW. Five new diagnoses were made at baseline (four TGW, one CGW) and only one during the follow‐up period in a TGW.

Among those living with HIV, 61% (*n* = 19) had a viral load (VL) <50 copies/mL with a median CD4 cell count of 848 cells/mm^3^ (IQR: 646–1075). Those with detectable VL had a median VL of 11,371 copies/mL (IQR: 235–24,210) with a median CD4 cell count of 305 cells/mm^3^ (IQR: 238–716). Among this group: 50% (*n* = 6) had discontinued treatment, 17% (*n* = 2) were on stable ART for more than 6 months, 17% (*n* = 2) had never initiated treatment, 8% (*n* = 1) had irregular adherence and 8% (*n* = 1) had started ART within the prior 6 months. ART was offered to those not on treatment (*n* = 13).

Among participants without HIV, 18.4% (*n* = 18) of CGW and 5.7% (*n* = 4) of TGW reported never having tested for HIV. Notably, 72.2% (*n* = 13) of CGW who had never been tested had been pregnant before, most (*n* = 11) more than once, despite routine antenatal screening in Argentina (National Law 25.543). Among those previously tested, the median time since the last HIV test was 5 months (IQR: 2–22) for TGW and 20 months (IQR: 8–59) for CGW (*p* = 0.001).

### 3.5 HIV PrEP Knowledge, Acceptance and Persistence

At baseline, only 16.7% (*n* = 16) of TGW and 3.1% (*n* = 3) of CGW were on PrEP. Among those not receiving PrEP, TGW reported higher rates of prior PrEP use (14.2% [*n* = 7] vs. 1% [*n* = 1]) and PrEP knowledge (50% [*n* = 24] vs. 18.8% [*n* = 18], *p* = 0.001) compared to CGW.

When offered PrEP, acceptance was higher in TGW than CGW (55.6% [*n* = 27] vs. 30.5% [*n* = 29]). Reasons for not initiating PrEP included: perceiving it as unnecessary (owing to low‐perceived risk or using another prevention method), fear of potential adverse events, not desiring taking medication and postponing PrEP for another time.

Regarding PrEP persistence, 69% (*n* = 49) of FSWs who started PrEP in this project discontinued during follow‐up (median time: 4.3 months, IQR: 1−10.5), with a higher discontinuation rate in CGW (22.5% [*n* = 22] in TGW vs. 46.5% [*n* = 28] in CGW).

### 3.6 Implementation of the Integrated SRH‐HIV Care Model

Early implementation of the comprehensive programme revealed several operational challenges, including missed entry visits, requiring methodological adaptations. Adaptive strategies included: extending recruitment periods to accommodate seasonal migration, engaging community leaders as trusted recruitment partners and expanding appointment availability for SRH services. Of the 101 CGW enrolled, 41 were referred to higher‐level facilities through the pathways for gynaecological care.

### 3.7 Satisfaction With “MAS por Nosotras”

At the 6‐month follow‐up, satisfaction with the care model and its package of services was notably high. Overall, 91.7% of participants rated the model as “very good” and 8.3% as “good.” Willingness to recommend the package to peers was similarly strong, with 78.5% reporting they were “very likely” and 15.7% “likely” to do so. Perceptions of adopting a comparable package within the public health system were more mixed but remained largely favourable, with half indicating they would be “very likely” to use it and 24.6% “likely.”

## 4 Discussion

We described the implementation of a community‐linked, gender‐focused integrated SRH‐HIV prevention and care model for FSWs in Buenos Aires. Building on formative research and co‐production alongside community‐based organizations (ATTTA and AMMAR) and the MoH, the model addressed gaps in SRH and HIV care access through a “one‐stop,” low‐barrier service delivery strategy, aligned with global recommendations for integrated care models [23]. Our findings provide preliminary evidence on the operational feasibility and acceptability of this integrated model in a primary care setting.

“MAS por Nosotras” involved two of the populations most affected by HIV in Argentina: transgender and cisgender FSWs. HIV prevalence in this cohort was high, especially in TGW (34% in TGW and 3.2% in CGW) [10, 15]. These results mirror regional and global data indicating that TGW experience the highest HIV burden worldwide, underscoring persistent structural barriers [24, 25]. Importantly, 18.4% of CGW reported never having been tested for HIV despite mandatory antenatal screening in Argentina (72.2% had at least one prior pregnancy), revealing either missed opportunities for routine screening or a lack of information about the test, a gap previously noted [26]. The proportion of the 37 FSWs living with HIV and detectable VL at baseline (39%), including prior ART discontinuation, irregular or no prior treatment, reveals persistent gaps in the care cascade. These findings are consistent with the wide variability in ART uptake (0%–100%) and adherence (50%–90%) reported in FSWs in the global literature [27, 28, 29]. Taken together, these contrasting patterns highlight the uneven landscape of HIV care among sex worker populations and underscore the need for strengthening HIV testing strategies within primary care and SRH services and for supporting engagement in care through community‐linked models [23, 28].

TGW reported higher baseline PrEP knowledge, prior use and acceptance than CGW, who showed limited prior exposure to PrEP and lower acceptance when offered in the integrated model (50%, 16.7%, 55.6% and 18.8%, 3.1%, 30.5%, respectively). These patterns are consistent with findings from South Africa and Brazil, where TGW have been the primary targets of PrEP programmes [30, 31]. However, the level of knowledge and acceptance observed in our TGW were slightly lower than the reported in those settings and lower than rates described in other studies [32, 33]. PrEP persistence was low (31%), compared to other studies that reported a 66%–83% retention at 6 months [34], with higher discontinuation among CGW, probably reflecting other priorities in their everyday life and specifically in health. Clinical trials similarly demonstrate low real‐world effectiveness with oral PrEP in CGW [35, 36]. These findings point to the need for education and differentiated PrEP strategies and demonstrate that our integrated model served as a key entry point for introducing PrEP to a population that has traditionally faced significant barriers to accessing national PrEP programmes.

As previously reported [37, 38] and identified in our formative research, FSWs face several barriers to access SRH and HIV healthcare, including anticipated external stigma in healthcare facilities, both related to gender identity and their occupation. Competing priorities—such as unstable housing, food insecurity, caregiving responsibilities and daily income generation—emerged as determinants that limit engagement in care, aligned with regional and global evidence showing inequities in HIV and SRH in women facing intersecting vulnerabilities [1, 2, 3, 4, 5, 6, 7, 26, 28]. These findings contextualize retention challenges and show that structural constraints outside the healthcare sector shape FSWs’ engagement in care. As WHO noted [23], navigating fragmented services further contributes to delay or avoid care, reinforcing the importance of integrated models that combine services in a single visit.

“MAS por Nosotras” successfully developed and delivered a comprehensive SRH‐HIV package of services—including HIV testing, treatment and prophylaxis; STIs care; mental health support; gender‐affirming care; and reproductive health with referrals—within an NGO research centre functioning as a primary care facility, aligned with other international experience of integrated models [39]. The implementation experience provides preliminary evidence regarding operational feasibility and acceptability among FSWs, despite their significant structural challenges such as high mobility, substance use, communication barriers (many lacking mobile phones), night‐time work schedules conflicting with morning appointments and competing economic priorities. Achieving a 66.5% retention rate at 6 months in this context underscores the value of adaptive operational strategies such as flexible scheduling, multiple follow‐up strategies and the sustained involvement of peer navigators. Although participant compensation and additional retention efforts within the research context may have contributed to follow‐up rates, several operational strategies used in the project could be adapted to non‐research public health settings. Community participation throughout the project was central, strengthening trust, reducing stigma and improving linkage to services, as proven in other countries of the region [28]. High levels of satisfaction and willingness to recommend “MAS por Nosotras” demonstrate that integrating multiple services into a single location meets the complex and overlapping SRH and HIV needs of FSWs. An in‐depth intersectional analysis is warranted to better understand how gender identity, migration, work context and other structural vulnerabilities interact to shape SRH‐HIV needs and outcomes, and to inform the adaptation and implementation of integrated models in other settings and for women in other situations of vulnerability.

This study has some limitations to be acknowledged. The study was performed in the city of Buenos Aires, aiming to reach FSWs living in the metropolitan area of Buenos Aires; results may not be generalizable to other regions. Recruitment through community‐based organizations may have overrepresented FSWs already connected with support networks, potentially underestimating unmet needs of those more socially isolated. The results are representative only of the study sample and may underreport the actual burden of disease in the broader FSW population. Additionally, the retention observed was suboptimal, which is consistent with challenges reported in other cohorts of hard‐to‐reach populations. While retention could have been higher, “MAS por Nosotras” successfully enabled 200 participants to receive a comprehensive health evaluation, providing care for more FSWs than would have occurred without this initiative. The overall project's duration did not allow for evaluation of longer‐term outcomes, nor implementation in other facilities, highlighting that expanded financial support is required to sustain longer follow‐ups. Challenging global and local socio‐economic and political contexts may pose additional barriers to expand or implement such care models. Still, the proposed package of services for this model was designed based on pre‐existing resources provided by the system, facilitating the potential sustainability and scaling up. Despite these limitations, the study provides valuable epidemiological data on SRH and HIV outcomes in a population underrepresented in national surveillance, while simultaneously piloting an integrated model of care that is uncommon in our setting.

## 5 Conclusions

“MAS por Nosotras” implemented a community‐linked, gender‐informed integrated SRH‐HIV model of care, articulated with community‐based organizations, the MoH and an NGO research centre. The model showed promising preliminary evidence regarding operational feasibility and acceptability, while facilitating access to HIV testing, PrEP, PEP and ART initiation or re‐engagement. The high prevalence of HIV—particularly among TGW—alongside low knowledge and PrEP use especially among CGW, and suboptimal viral suppression among individuals living with known HIV, highlight the need to enhance HIV prevention and treatment strategies for FSWs.

“MAS por Nosotras” is one of the first studies to provide comprehensive information on FSWs’ health in Argentina and proposes an evidence‐based care model that could guide public health efforts to improve care of other women in vulnerable situations. By incorporating community leadership, peer navigation, intersectional perspectives and coordinated referral pathways, “MAS por Nosotras” demonstrates a promising strategy for expanding equitable SRH‐HIV services in Argentina and Latin America.

## Author Contributions

*Conceptualization*: VF, IA and SW. *Data curation*: MMS and VZ. *Formal analysis*: MMS, AZ, VZ, IA and AN. *Funding acquisition*: VF, IA, AD and SW. *Investigation*: MMS, VZ, EF, AZ, NC, CFP and MVI. *Methodology*: VF, IA, AD, GO, MR, SW and AN. *Supervision*: VF, IA, MIF and SW. *Writing – original draft preparation*: MMS. *Writing – review and editing*: MMS, IA, VZ, VF and SW. *Final review and approval*: MMS, IA, VZ, SV, MVI, AA, EF, ACZ, AN, NFC, MR, GO, CFP, MIF, AD, SW and VF.

## Funding

This project was funded under Women's health and economic empowerment for a COVID‐19 Recovery that is Inclusive, Sustainable and Equitable (Women RISE), an initiative of the International Development Research Centre, the Canadian Institutes of Health Research, and the Social Sciences and Humanities Research Council. Grant number: 110045.

## Conflicts of Interest

The authors declare that the research was conducted in the absence of any commercial or financial relationships that could be construed as a potential conflict of interest.

## Supporting information




**Table S1**: Description of the procedures performed at the visits.

## Data Availability

The authors will make, upon request, without undue reservation, the raw data that support the conclusions of this article.
