# Supplementary material for: Integrating HIV Into Sexual and Reproductive Health Services for Female Sex Workers in Buenos Aires, Argentina: A Comprehensive Model Designed Through Intersectional Collaboration
Source: J Int AIDS Soc. 2026 Jul 25;29(Suppl 3):e70167. doi: 10.1002/jia2.70167 (PMC13401715; doi:10.1002/jia2.70167)
Supplement: Supplementary file 1 — Table S1: Description of the procedures performed at the visits. [file JIA2-29-e70167-s001.docx]

**Supplementary Material**

**Table 1. Description of the procedures performed at the visits**

| **Procedure** | **Description** |
| --- | --- |
| Informed consent | Sign of the informed consent form. |
| Clinical evaluation | Medical history; medication use; history and treatment of STI; STI prevention methods; vaccination history; COVID-19 diagnosis and vaccination status. |
| HIV rapid test | Dual (both HIV and syphilis) or simple (only HIV) rapid test (Abbott® Determine/Bioline).  HIV diagnosis was performed according to national guidelines. |
| Counseling and offer of PEP and PrEP | According to risk defined per national guidelines. |
| STI screening | Serologies for HIV, syphilis and viral hepatitis (A, B, C); anal, cervical (CGW), urinary (TGW), and oropharyngeal swabs for *Neisseria gonorrhoeae* (NG) and *Chlamydia trachomatis* (CT). |
| STI counseling and offer of condoms | Counseling about safe sexual practices and offering prevention methods. |
| Laboratory tests | Blood count, liver function tests, lipid profile, glucose, creatinine and urea; CD4 cell count and HIV viral load (when clinically indicated). |
| Vaccines | Vaccines delivered at the visit according to age, comorbidities, and vaccination history. |
| Gynaecological assessment | Family planning (pregnancies and abortions); contraceptive use and preferences; pregnancy test (when clinically indicated); cervical cytology and hrHPV genotyping (16, 18, 31, 33, 35, 39, 45, 51, 52, 53, 56, 58, 59, 66, 68); mammography and/or breast ultrasonography according to age and family history. Assessments at six-month visit were performed according to baseline results. |
| Proctological assessment | Previous proctological evaluations; anal cytology and hrHPV genotyping (16, 18, 31, 33, 35, 39, 45, 51, 52, 53, 56, 58, 59, 66, 68); high resolution anoscopy (when clinically indicated).  Assessments at six-month visit were performed according to baseline results. |
| Hormone gender-affirming therapy | Endocrinology evaluation and hormonal dosage. |
| Psychosocial interview | Sociodemographic and economic characteristics; sex work context; health-service use; sexual and reproductive health; depressive symptoms (CES-D), suicidal ideation screener, post-traumatic stress disorder (PCL-5), substance use (DAST-10), alcohol use (AUDIT), tobacco use, resilience (CD-RISC-10), quality of life (SF-12), and social support. Sex work stigma (ESWS), transgender identity stigma (TIS) and HIV stigma (Berger Scale). Intimate partner and sexual violence (WHO Intimate Partner Violence Severity Scale). |
| Satisfaction and structural priorities assessment | Satisfaction with the care model; willingness to recommend the model to peers; willingness to use a similar package within the public health system; ranking of structural priorities (e.g., housing, financial stability, caregiving responsibilities, SRH needs). |
| Psychological evaluation | Only performed when significant psychopathological symptoms or suicidal risk were identified during psychosocial interviews. |

Abbreviations: STI, sexually transmitted infection; HIV, human immunodeficiency virus; PEP, post-exposure prophylaxis; PrEP, pre-exposure prophylaxis; CGW, cisgender woman; TGW, ransgender woman; hrHPV, high-risk human papillomavirus; SHR, sexual and reproductive health.
